# Bifurcation Oscillator as an Advanced Sensor for Quantum State Control

**DOI:** 10.3390/s22176580

**Published:** 2022-08-31

**Authors:** Dmitrii Pashin, Marina Bastrakova, Arkady Satanin, Nikolay Klenov

**Affiliations:** 1 Faculty of Physics, Lobachevsky State University of Nizhny Novgorod, 603950 Nizhny Novgorod, Russia; 2 Russian Quantum Center, 143025 Moscow, Russia; 3 Higher School of Economics, Russia National Research University, 101000 Moscow, Russia; 4 Dukhov All-Russia Research Institute of Automatics, 101000 Moscow, Russia; 5 Faculty of Physics, Lomonosov Moscow State University, 119991 Moscow, Russia; 6 Science and Research Department, Moscow Technical University of Communication and Informatics, 111024 Moscow, Russia

**Keywords:** nonlinear oscillator, qubit, bifurcation, measurement of superposition qubit states, entanglement

## Abstract

We study bifurcation behavior of a high-quality (high-Q) Josephson oscillator coupled to a superconducting qubit. It is shown that the probability of capture into the state of dynamic equilibrium is sensitive to qubit states. On this basis we present a new measurement method for the superposition state of a qubit due to its influence on transition probabilities between oscillator levels located in the energy region near the classical separatrix. The quantum-mechanical behavior of a bifurcation oscillator is also studied, which makes it possible to understand the mechanism of "entanglement" of oscillator and qubit states during the measurement process. The optimal parameters of the driving current and the state of the oscillator are found for performing one-qubit gates with the required precision, when the influence on the qubit from measurement back-action is minimal. A measurement protocol for state populations of the qubit entangled with the oscillator is presented.

## 1. Introduction

Scalable quantum computing and quantum error correction require high-precision projective measurements of qubit states at scales shorter than coherence time. In superconducting systems, measurements are usually based on qubit-microwave resonator coupling (dispersive readout) [1,2,3,4,5]. The disadvantage of this method, associated with a fast decay rate of the qubit state due to coupling with the environment through a resonator (Purcell effect), is currently successfully overcome using Purcell filters [6,7]. Another development of methods of dispersive readout of qubits is to use a nonlinear element: multimode resonators [8,9], Josephson parametric [10,11,12] and bifurcation amplifiers [13,14,15,16,17,18,19,20].

Such nonlinear “sensors” are based on the properties of a hysteresis bistable system with metastable dynamics. Using this approach, single-shot readout of transmon qubits states with high fidelity ∼94% was experimentally demonstrated [13,14]. Note that the first experiments on the use of Josephson bifurcation amplifiers were carried out at low *Q*-factors [15,16]; therefore, switching occurred between various stable oscillations because of noise with decrease of the qubit lifetime. Subsequently, significant progress in coherence control was achieved using high-Q quantum electrodynamics schemes [4,21] when embedding Josephson nonlinear amplifiers in coplanar waveguides (cavity Josephson bifurcation amplifier) [13,17]. Switching between bistable states in these experiments occurs due to amplitude scanning of the drive current up to an operating value. It is important that, when choosing this working range of amplitudes, one of the main criteria is the existence of a bifurcation behavior of the measuring oscillator for only one qubit basis state. Following this approach, it is not possible to implement single-shot readout for qubit superposition states. Note that such systems have been actively used in recent years as highly sensitive detectors in hybrid systems [22,23,24,25], as well as for observing nonlinear effects [26,27,28,29,30,31,32,33,34,35].

In this paper, we propose a new measurement method for superpositions of qubit states using the discriminating property of a bifurcation Josephson oscillator. It is based on the sensitivity of the probability of capture into either metastable equilibrium states of the driving nonlinear oscillator, rather than on a change in the switching probability, as was assumed in the papers [13,17,30]. In the classical approximation, metastable equilibrium states are realized dynamically under the action of the drive current (as in the case of the Duffing oscillator [36]). These states are separated in phase space by a separatrix. To describe the selective behavior of the device—mesoscopic Josephson oscillator, it is necessary to take into account a sufficiently large number of its states, so as to realize the passage of the system through the energy region where the separatrix is located in the classical approximation. Note that the proposed measurement method does not require amplitude scanning, and a high-Q measuring oscillator can be used in the parameter range where bifurcation behavior exists for both qubit basis states.

We considered a quantum analogue of the oscillator bifurcation behavior in order to understand the mechanism of an "entanglement" of the oscillator and qubit states. A measurement protocol was proposed and a Rabi-based-procedure for a possible experiment on initialization of a qubit and measuring oscillator states due to a modulated pulsed current was numerically simulated. The influence of the measuring oscillator on the process of qubit initialization by Rabi pulses is studied.

## 2. Model and Basic Equations

**Figure 1:**
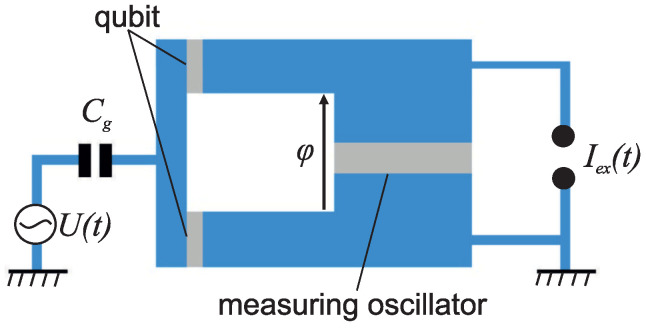
Sketch of a quantronium qubit coupled to a measuring oscillator.

The prototype of the measured qubit is the “quantronium” system [16,37]. The quantronium qubit (see Figure 1) is a split Cooper pair box with two Josephson junctions (each is characterized by Josephson $E_{J}/2$ and charging $E_{C}$ energies). The qubit states can be initialized by applying a control voltage U(t) through a capacitor $C_{g}$.

The measuring oscillator is a Josephson junction with Josephson energy $E_{J}^{R}\;\gg \;E_{J}$, to which the drive current $I_{ex}\left(t\right)$ is applied. The Hamiltonian of the system reads

(1) $$\begin{array}{cc} \; & H\;=\;H_{q}\;+\;H_{osc}\;+\;H_{int}, \end{array}$$

where the Hamiltonian of the qubit with the control field $\epsilon \left(t\right)\;=\;2E_{c}C_{g}U\left(t\right)/e$ can be written as follows:

(2) $$H_{q}\;=\;-\frac{1}{2}\left(\epsilon \left(t\right)\sigma_{x}\;+\;E_{J}\sigma_{z}\right),$$

where $\sigma_{x},\;\sigma_{z}$ are Pauli matrices. In the Hamiltonian of the measuring oscillator, the generalized coordinate is the phase difference at the Josephson junction φ:

(3) $$H_{osc}\;=\;C\Phi_{0}^{2}\frac{{\dot{\varphi }}^{2}}{2}\;-\;E_{J}^{R}\cos\varphi \;-\;\Phi_{0}I_{ex}\left(t\right)\varphi ,$$

where *C* is the junction capacitance, $\Phi_{0}\;\equiv \;\hbar /\left(2e\right)$ is the magnetic flux quantum, and $I_{ex}\left(t\right)$ is the external control current. The interaction of a qubit and an oscillator included in one superconducting circuit is determined by the constant $\lambda \;=\;E_{J}/8E_{J}^{R}$:

(4) $$H_{int}\;=\;\frac{\lambda }{2}E_{J}^{R}\left(\varphi^{2}\;-\;\frac{\varphi^{4}}{48}\right)\sigma_{z}.$$

The control Rabi pulse U(t) makes it possible to create the required superposition of qubit states. Below we describe a new scenario of states readout using the simple manipulations with the drive current $I_{ex}\left(t\right)$ applied to the Josephson oscillator.

## 3. The Main Idea: Measuring a Qubit with a High-Q Nonlinear Oscillator

It is well known that a nonlinear oscillator (3) driven by a periodic external current $I_{ex}\left(t\right)\;=\;I_{0}\cos\left(\omega t\right)$ and operating in a weak nonlinear regime can be in two dynamic equilibrium states with different amplitudes and phases [36]. Let us briefly describe the change in the phase portrait of the oscillator during its interaction with a qubit.

In the slowly varying amplitude approximation [38,39], the dynamic equilibrium states correspond to stable equilibrium points on the phase plane, separated by a separatrix. In the weakly nonlinear regime, the Hamiltonian for the new slowly varying variables (q, p) (see Appendix A) is given as

(5) $${\bar{H}}_{osc}\;=\;\hbar \left(\omega_{0}\;-\;\omega \right)\left(\frac{q^{2}}{2}\;+\;\frac{p^{2}}{2}\right)\;-\;\beta {\left(\frac{q^{2}}{2}\;+\;\frac{p^{2}}{2}\right)}^{2}\;-\;fq,$$

where $\omega_{0}\;\equiv \;\sqrt{\frac{E_{J}^{R}}{C\Phi_{0}^{2}}}$ is the natural frequency of the measuring oscillator, $\beta \;\equiv \;\frac{e^{2}}{4C}$ is the nonlinearity parameter and $f\;\equiv \;I_{0}\sqrt{\frac{\hbar }{4\omega_{0}C}}$ is the driving force amplitude. For numerical calculations, we will use the parameter values close to the experimental ones [16,24]:

(6) $$\begin{array}{ccccc} \frac{\omega_{0}}{2\pi }\;=\;0.16\;\mathrm{GHz}, & \;\omega \;=\;0.95\omega_{0}, & \;\beta \;=\;1.8\;\times \;10^{-4}\hbar \omega_{0}, & \;f\;=\;0.05\hbar \omega_{0}, & \;\lambda \;=\;0.03. \end{array}$$

**Figure 2:**
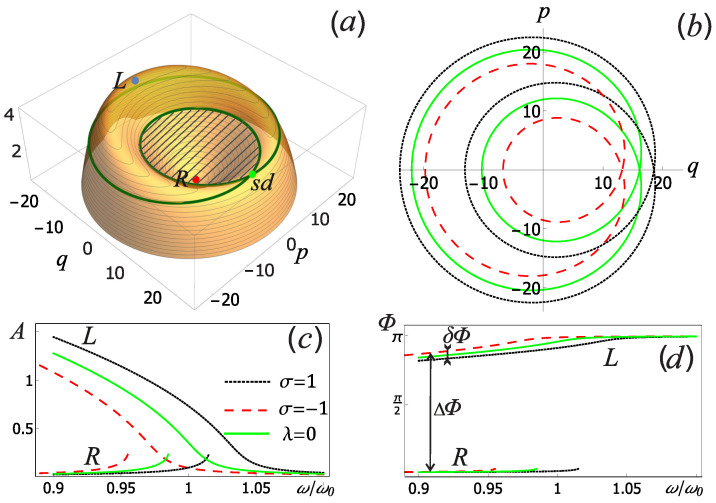
(**a**) The quasi-energy surface ${\bar{H}}_{osc}\left(q,\;p\right)$ is represented in units of $\hbar \omega_{0}$. The capture area in the right equilibrium point corresponds to the shaded area $S_{R}$. The left and right equilibrium points are denoted by the symbols *L* and *R*, respectively, and sd is the saddle point. The separatrix (**b**), amplitude (**c**) and phase (**d**) of the stable oscillations of the nonlinear oscillator as a function of the drive current frequency for different qubit states (σ = ±1). We assume that there was no connection with the qubit when the coupling parameter is equal to λ = 0. Other parameters are determined by the relations (6).

The resulting Hamiltonian (5) is time-independent, and the corresponding quasienergy surface is shown in Figure 2a. *L* and *R* symbols denote the left and right stable focuses, which are in the corresponding extrema of the quasienergy surface, and the letters sd denote the saddle point characterizing the separatrix.

For a certain range of initial conditions located inside the lobes of the separatrix and under weak dissipation (large resonator quality factor *Q*), the deterministic process of capture into one of the equilibrium points occurs. Another capture scenario takes place when initial conditions are given outside the separatrix. In this case, the oscillator is captured into either the *L* focus or the *R* focus randomly. As shown in [39], the capture probability is determined by the area that sweeps out by the separatrix in phase space around the region that corresponds to the considered equilibrium point. The result obtained is described by the well-known Arnold formula [40]:

(7) $$P_{\alpha }\;=\;\frac{S_{\alpha }}{S_{L}\;+\;S_{R}},\;\alpha \;=\;L,\;\;R,$$

where $S_{L}$ and $S_{R}$ are the areas of the separatrix lobes. As can be seen from (7), the capture probability does not depend on specific initial conditions outside the separatrix.

The idea of the proposed measurement method: the coupling of the measuring nonlinear oscillator with the qubit induces a dependence of the capture probability into one of the equilibrium points (for example, in the right one–$P_{R}$) on the qubit state. Since the two stable oscillations of the measuring oscillator differ from each other in phase, the probabilities $P_{R}$ and $P_{L}$ can be measured experimentally.

Note that the phase difference for two stable oscillations in a measuring circuit has recently been widely used for dispersive quantum measurements [13,17,22,23,24], where the switching probability between the two stable processes is measured, rather than the capture probability. These methods show the highest discrimination power in the case when one of the stable oscillations disappears at the operating value of the amplitude *f* and the frequency ω of the external force for one qubit basis state. However, in the range of parameters, when there are two stable oscillations for both qubit basis states, the discrimination power of the measuring oscillator with high quality factor (several hundreds) will be extremely low. The method proposed in this paper makes it possible to carry out dispersive measurements of qubits in this parameters range, refusing to scan by the amplitude of the drive current.

To begin with, we will assume that when the control field ϵ(t) = 0 the qubit may be in one of the basis states with quantum number σ = ±1 (which is determined by the eigenvalue of the Pauli matrix $\sigma_{z}$). Then the oscillator Hamiltonian reduces to two independent Hamiltonians $H^{\pm }$ corresponding to the ground | ↑〉 and the excited | ↓〉 qubit states (“pseudospin” polarization), which are obtained from Equation (5) by replacing $\omega_{0}\;\to \;\omega_{0}^{\pm }\;\equiv \;\omega_{0}\left(1\;\pm \;\frac{\lambda }{2}\right)$ and $\beta \;\to \;\beta^{\pm }\;\equiv \;\beta \left(1\;\pm \;\frac{\lambda }{4}\right)$. Since the qubit state affects the natural frequency and the nonlinearity of the measuring oscillator, it also changes the separatrix in phase space, as shown in Figure 2b. The stable oscillations of the nonlinear oscillator in the slowly varying amplitude approximation, corresponding to the points *L* and *R* in Figure 2a, can be characterized by the amplitude *A* and the phase Φ, which are found for different polarization as $A_{\pm }\;=\;\sqrt{\frac{\hbar \omega_{0}}{E_{J}^{R}}\left(q_{\pm }^{2}\;+\;p_{\pm }^{2}\right)}$, where $q_{\pm }$ and $p_{\pm }$ correspond to the coordinates of *L* or *R* equilibrium points in Figure 2a for each qubit basis state. The oscillation phase for the left equilibrium point is $\Phi_{\pm }\;=\;\pi \;+\;\mathrm{acrtg}\left(\frac{p_{\pm }}{q_{\pm }}\right)$, for the right equilibrium point is $\Phi_{\pm }\;=\;\mathrm{acrtg}\left(\frac{p_{\pm }}{q_{\pm }}\right)$. Figure 2c,d shows the dependences of the amplitudes and phases of stable oscillations of the measuring oscillator on the external force frequency at the quality factor of Q = 500, from which the operating frequency range is clearly visible.

**Figure 3:**
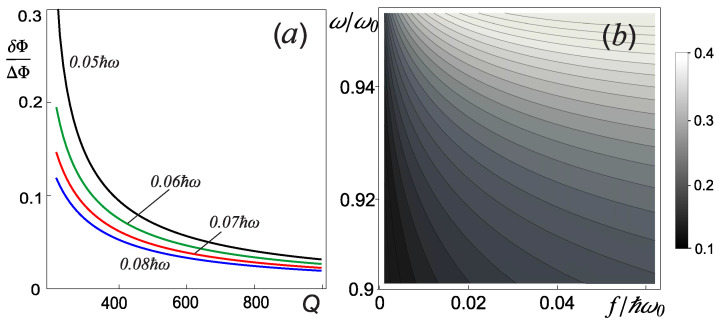
(**a**) The value of δΦ/ΔΦ as the function of the quality factor *Q* of the nonlinear oscillator for different values of the drive current amplitude *f*: 0.05ℏω (black), 0.06ℏω (green), 0.07ℏω (red) and 0.08ℏω (blue). (**b**) The dependence of Δ on the drive current *f* amplitude and frequency ω. Other parameters of the system are determined by the relations (6).

Due to the fact that the proposed measurement method is based on measuring the phase difference of various stable oscillations of the measuring oscillator ΔΦ (see Figure 2d), it is necessary that such a difference be much larger than the phase perturbation δΦ introduced due to the different qubit states (ΔΦ ≫ δΦ). Figure 3a shows that such a condition is not satisfied at a low Q-factor in the parameters range considered. In addition, we note that when measuring the results of quantum single-qubit operations, it is necessary to carry out multiple measurements and collect statistics (capture probabilities) to achieve the required precision. To estimate the number of single measurements, we analyzed the influence of the control current parameters on the efficiency, Δ, of separating qubit states with different quantum number σ = ±1. Each qubit basis state will be characterized by the capture probability of the measuring oscillator into one of the equilibrium points (for example, into the right one) $P_{R}^{\pm }$. Then the separation efficiency can be defined as $\Delta \;=\;P_{R}^{+}\;-\;P_{R}^{-}$. Figure 3b shows the dependence of this quantity on various parameters of the drive current of the measuring oscillator. It can be seen that for any frequency ω, there is an optimal amplitude *f*. Based on the analysis of Figure 3b, the optimal parameters of the drive current (6) were chosen.

We stress again, that the key element in the proposed measurement method is the passage of the separatrix in phase space by the system. This is equivalent to the passage of separatrix energy on the quasi-energy surface (Figure 2a). Near this energy the quasi-classical description is broken [41], and the temperature is assumed to be sufficiently low $T\;<\;\hbar \omega_{0}/k_{B}\;\approx \;0.1$ K; therefore, a quantum description of the system is necessary. In terms of the creation and, annihilation operators for the measuring oscillator, and for the time when the control field has already been turned off, ϵ(t) = 0, the Hamiltonian of the system can be written as [16]

(8) $$H\;=\;H_{q}\;+\;\hbar \omega_{0}\left(1\;+\;\frac{\lambda }{2}\sigma_{z}\right)a^{†}a\;+\;\lambda \frac{\hbar \omega_{0}}{4}\sigma_{z}\left(a^{†2}\;+\;a^{2}\right)-\frac{\beta }{6}\left(1\;+\;\frac{\lambda }{4}\sigma_{z}\right){\left(a^{†}\;+\;a\right)}^{4}\;-\;\sqrt{2}f\left(a^{†}\;+\;a\right)\cos\left(\omega t\right),$$

with the commutation relation $[a,\;a^{†}]\;=\;1$.

In the rotating wave approximation (Appendix A), the system Hamiltonian becomes time-independent:

(9) $$H_{rwa}\;=\;H_{q}\;+\;\hbar \left(\omega_{0}\left(1\;+\;\frac{\lambda }{2}\sigma_{z}\right)\;-\;\omega \right)a^{†}a-\beta \left(1\;+\;\frac{\lambda }{4}\sigma_{z}\right)aa^{†}a^{†}a\;-\;\frac{f}{\sqrt{2}}\left(a^{†}\;+\;a\right).$$

The quasistationary states $|\;\psi_{i}\rangle$ of the system are determined by the equation:

(10) $$H_{rwa}\;|\;\psi_{i}\rangle \;=\;E_{i}\;|\;\psi_{i}\rangle .$$

As in the classical case for σ = ±1 in the Equation (9), there are formally two subsystems corresponding to two qubit states. These subsystems evolve independently and are characterized by different Hamiltonians $H^{\pm }$:

(11) $$H^{\pm }\;=\;\hbar \left(\omega_{0}^{\pm }\;-\;\omega \right)a^{†}a\;-\;\beta^{\pm }aa^{†}a^{†}a-\frac{f}{\sqrt{2}}\left(a^{†}\;+\;a\right).$$

Then instead of (10) we can write:

(12) $$H^{\pm }\;|\;\phi_{j}^{\pm }\rangle \;=\;E_{j}^{\pm }\;|\;\phi_{j}^{\pm }\rangle .$$

The Hilbert space of two-component basis functions $\left\{|\;\psi_{i}\rangle \right\}$ splits into two independent subspaces with bases $\{|\;\phi_{j}^{+}\rangle \;⊗\;|↑\rangle \}$ and $\{|\;\phi_{j}^{-}\rangle \;⊗\;|↓\rangle \}$, respectively. These basis functions can be easily found by numerical methods.

The case λ = 0 was investigated in [39], where dissipation was taken into account and it was shown that for a quantum system with two attractors (which can be considered as an analogue of potential wells), the quantum Arnold formula for the capture probabilities in the attractors can be obtained. In the semiclassical approximation, the generalized formula follows from the Bohr-Sommerfeld quantization condition, according to which the area swept by the trajectory of a particle moving in a potential well is proportional to the number of levels in the well. When the number of levels near the separatrix energy is much less than the number of levels related to the left or right equilibrium points, we can get a simple analogy that the ratio between the areas of separatrix lobes in (7) is determined by the number of levels.

It can be seen from (9) and (11), that the interaction (λ ≠ 0) of the oscillator with the qubit leads to the splitting of the levels into two series corresponding to different polarizations of the qubit. At the same time, the probability of capture into series of doubling attractors will also depend on the polarization of the qubit. It means that the Josephson oscillator can operate as a sensor of quantum systems. In the next section, the selective characteristics of the proposed sensor will be analyzed in more detail.

## 4. The Measurement Procedure for Superpositions of Basis Qubit States

Here we consider the dynamics of the qubit coupled with the measuring nonlinear oscillator, paying special attention to its potential as a sensor for measuring superposition states of the qubit. We take into consideration the measuring oscillator dynamics in mesoscopic (quantum) mode of operation in order to minimize back action (the influence of the sensor on the qubit states).

**Figure 4:**
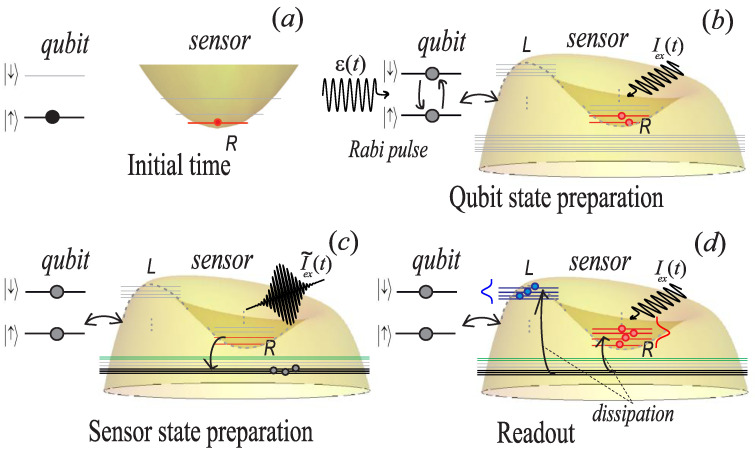
Schematic representation of the measurement procedure. (**a**) Initially, we assume that the qubit and the sensor are in the ground state. There is no bifurcation behavior of the sensor because no control current is applied to it. (**b**) The qubit is initialized by a Rabi pulse. There is back action on the sensor due to the coupling. The sensor control current is on. (**c**) The sensor is excited to the levels corresponding to the region outside the lobes of the classical separatrix by an additional pulsed current. (**d**) The sensor relaxes into a superposition of levels corresponding to the right and left equilibrium points. The corresponding probabilities depend on the qubit state.

Our original scheme, see Figure 4, for measuring qubit states has three stages: (1) qubit state initialization based on microwave technique; (2) preparation of the sensor state (output outside the separatrix); (3) readout of qubit states by the nonlinear oscillator. In the following, we will discuss each stage in detail.

*Qubit state preparation.* At the initial moment, t = 0, we believe that the system of “qubit + nonlinear sensor” is initialized near their ground states (Figure 4a). The state of the this system is factorized ∣ Ψ(0)〉 =∣ Φ(0)〉 ⊗ ∣ q(0)〉, where ∣ Φ(0)〉 is the initial state of the measuring oscillator, and ∣ q(0)〉=| ↑〉 is the initial qubit state. Then the process of preparation (recording) is carried out qubit states using the control field ϵ(t), see the diagram in Figure 4b. Neglecting the relaxation processes during qubit preparation, the evolution of the system (8) can be found by solving the nonstationary Schrödinger equation.

Since the qubit continuously interacts with the measuring oscillator, it is necessary to analyze the influence of this interaction on the qubit (back action effect). To do this, we define the fidelity, *F*, of preparing qubit states by the Rabi pulse, according to [42]:

(13) $$F\;=\;\frac{1}{6}\sum_{\alpha }\mathrm{Tr}\left(\rho_{\alpha }\cdot \rho_{\alpha }^{0}\right),$$

where $\rho_{\alpha }\;\equiv \;{\mathrm{Tr}}_{ocs}\left(\rho \right)$ is the reduced density operator for the qubit subsystem at the end of the Rabi pulse at the initial state of the qubit ∣ α〉, and $\rho_{\alpha }^{0}$ is the density matrix of the qubit after the pulse, but without taking into account the connection with the oscillator (λ = 0). The summation in (13) occurs over the six initial states of the qubit ∣ q〉 = ∣ α〉: $|↓\rangle$, $|↑\rangle$, $\frac{|↓\rangle \pm |↑\rangle }{\sqrt{2}}$ and $\frac{|↓\rangle \pm i|↑\rangle }{\sqrt{2}}$. It is obvious that the fidelity, *F*, depends not only on the parameters of the qubit control field, but also on the initial state of the measuring oscillator due to the connection of subsystems, which inevitably gives rise to entanglement of their states.

The von Neumann entropy (an entanglement measure of subsystems) was calculated:

(14) $$S\;=\;-\eta_{+}\ln\eta_{+}\;-\;\eta_{-}\ln\eta_{-},$$

where $\eta_{\pm }\;=\;\frac{1}{2}\left(1\;\pm \;\left|s\right|\right)$. Note that the entanglement of the system is determined only by the length of the Bloch vector $s\;=\;\mathrm{Tr}(\vec{\sigma }\rho_{\alpha })$ [43].

**Figure 5:**
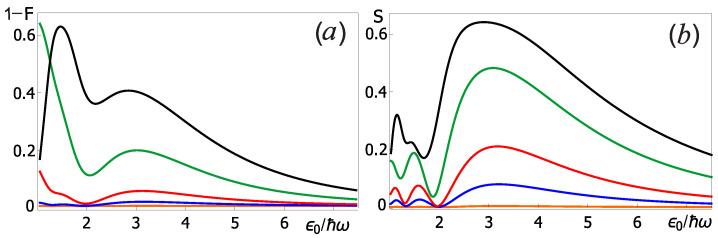
The infidelity (**a**) and the von Neumann entropy (**b**) of the system at the end of the Rabi pulse as a function of the amplitude of the qubit control field $\epsilon_{0}$ for various occupation numbers of the measuring oscillator initial state *n*: 0 (orange), 5 (blue), 10 (red), 20 (green), and 30 (black). Other parameters of the system are determined by the relations (6).

Figure 5 shows the results of the numerical calculation of the infidelity 1 − F and the von Neumann entropy *S* for different the 2π-pulse amplitude and initial states of the measuring oscillator averaged over the Pauli eigenstates. It can be seen from the analysis of this figure that the infidelity, 1 − F, decreases with increasing 2π-pulse amplitude $\epsilon_{0}$. This can be explained by the fact that the duration of the 2π-pulse decreases with increasing amplitude $\tau_{R}\;∼\;1/\epsilon_{0}$, and, consequently, the interaction time of the qubit with the measuring oscillator also decreases. However, we are limited by the range of operating control pulse amplitudes, for which we can neglect leakage since the quantum system leaves the two-level qubit subspace. Note also that in Figure 5a there is a clear local minimum near the value $\epsilon_{0}\;=\;2\hbar \omega$; at this pulse amplitude the Rabi frequency is equal to the natural measuring oscillator frequency. Qualitatively similar behavior was also observed for the von Neumann entropy.

**Figure 6:**
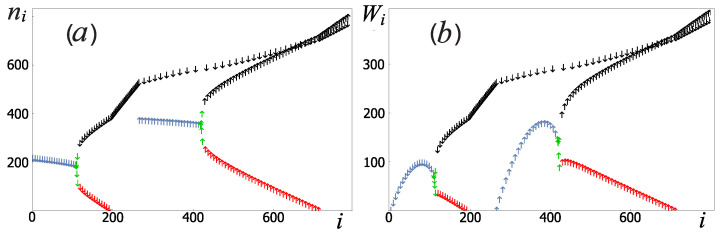
(**a**) Dependence of the average occupation number of the measuring oscillator $n_{i}\;=\;\langle \psi_{i}\;|\;a^{†}a\;|\;\psi_{i}\rangle$ as a function of the Hamiltonian (10) level numbers. The numbering of levels for each qubit state is chosen in descending order by energy (more details in [39]). The symbols ↑ and ↓ denote the system levels in which the qubit is in the ground state $|↑\rangle$ and the excited state $|↓\rangle$, respectively. (**b**) The dependence of the probability of leaving the level *i* per unit time $W_{i}$ in units of $\frac{2\pi g\kappa^{2}}{\hbar^{2}}$ (The probability of leaving the level $W_{i}$ will be defined strictly on the next section). Four levels with values near $W_{i}\;=\;0$ correspond to the classical equilibrium points. The groups of levels related to the left and right equilibrium points (see Figure 2a) are shown in blue and red color, green color indicates the levels corresponding to the energy of the separatrix, and black color indicates the levels corresponding to the classical region in the phase space outside the separatrix. The system parameters are determined by the relations (6).

Another important result is that 1 − F and *S* grow with an increase in the occupation number of the initial state of the measuring oscillator, see series of curves in Figure 5. Therefore that effective control (with minimal back action effect) of the qubit by pulses is possible only when the measuring oscillator is in a superposition of states with a small value of the occupation number *n*. Note that the acceptable fidelity of performing single qubit operations ($1\;-\;F\;<\;10^{-3}$) at the optimal amplitude $\epsilon_{0}\;=\;2\hbar \omega$ is achieved for the measuring oscillator initial states, at which n ⩽ 5. Analyzing the average occupation number of the measuring oscillator for each system level number, see Figure 6a, one can see that the necessary oscillator states (n ⩽ 5) are close to the level corresponding to the right equilibrium point for each qubit basis state (red color in Figure 6a). The initialization of the measuring oscillator to these levels can be realized, for example, by adiabatically slow switching on of the drive current, since at f = 0 there is only the right equilibrium point. In the classics, this case corresponds to the fact that the area $S_{L}$ in the Formula (7), related to the left equilibrium point, vanishes.

**Figure 7:**
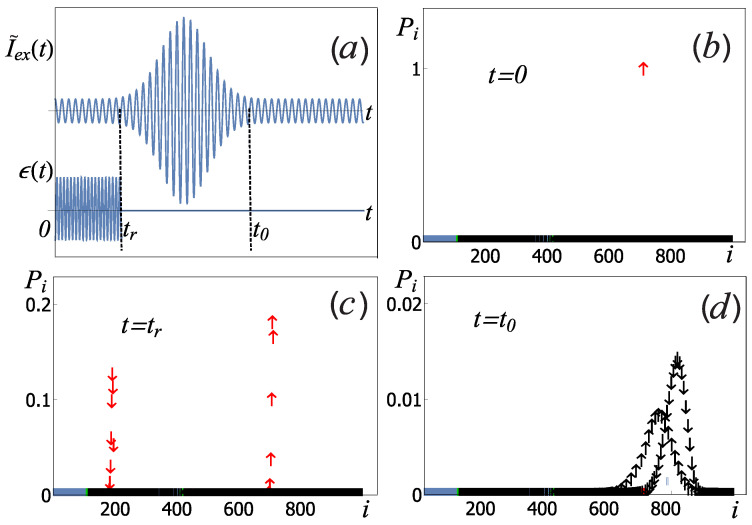
(**a**): The diagram of the sequence of pulses acting on the qubit ϵ(t) and on the sensor ${\tilde{I}}_{ex}\left(t\right)$. The recording Rabi pulse for the qubit has the duration of $t_{r}$. After this pulse, $t_{r}\;<\;t\;<\;t_{0}$, the modulating pulse is applied to the sensor. The values of the diagonal elements of the system density matrix $P_{i}$, depending on the level number *i*, under the sequential action of the π/2 pulse on the qubit and the pulse current on the oscillator at different times: (**b**) at the initial moment of time t = 0, (**c**) at the end of the Rabi pulse $t\;=\;t_{r}$, (**d**) after initialization of the measuring oscillator $t\;=\;t_{0}$. The use of colored arrows is the same as in Figure 6a,b. Numerical calculations are made for $\epsilon_{0}\;=\;1.2\hbar \omega_{0}$, $I_{p}\sqrt{\frac{\hbar }{4\omega_{0}C}}\;=\;3.9\hbar \omega_{0}$, and $\tau \;=\;5.5\frac{1}{\omega_{0}}$, other system parameters are determined by the relations (6).

*Preparation of the sensor state*. After the qubit preparation, it is necessary to excite the oscillator to levels corresponding to the classical region outside the separatrix (indicated in black in Figure 6a). A schematic representation of this process is shown on the Figure 4c. One way of such excitation is to use an additional short modulating pulse with a carrier frequency ω equal to the frequency of the drive current [44]. The total external current will be written as ${\tilde{I}}_{ex}\left(t\right)\;=\;I_{ex}\left(t\right)\;+\;I_{p}e^{-\frac{{\left(t-t_{p}\right)}^{2}}{2\tau^{2}}}\cos\left(\omega t\right)$, where $t_{p}\;=\;\left(t_{0}\;-\;t_{r}\right)/2$. This pulse does not change the expectation value $\langle \sigma_{z}\rangle$ of the qubit, since at this stage the action of the qubit control pulse ended ϵ(t) = 0, and $[\sigma_{z},\;H_{rwa}]\;=\;0$. The sequence of applying signals to the qubit and the sensor is shown in Figure 7a. Note that in the classical consideration, the process of capturing the measuring oscillator into one of the equilibrium points is considered to be completed immediately after the system enters the region in the phase space inside the separatrix. In this case, the process of transition between the regions of the separatrix belonging to different equilibrium points is impossible. In the quantum case, levels that are close to the energy corresponding to the classical separatrix cannot be strictly assigned to one of the equilibrium points due to the tunneling effect [39], but as one moves away from the separatrix energy, some levels are more and more localized near the classical equilibrium points. If there are sufficiently many such levels, then for levels that are far from the separatrix energy, the tunneling effect becomes negligible. Due to this, in the process of relaxation, transitions between groups of levels related to different equilibrium points can be neglected.

Figure 7b–d shows the results of numerical simulation of the qubit initialization by a π/2-pulse and excitation of the measuring oscillator by the modulated current pulse ${\tilde{I}}_{ex}\left(t\right)$. At the initial time, Figure 7b, the system was in the state $|\;\Phi \left(0\right)\rangle \;⊗\;|↑\rangle$, where the oscillator wave function ∣ Φ(0)〉 corresponds to the right equilibrium point with a small average occupation number (red color in Figure 6a). Usually, in order to create a superposition state of a qubit, they tend to reduce the impact of the measuring device on the qubit during the recording process. In our case, this would be possible if the qubit-oscillator coupling parameter λ = 0. In this case, the wave function of the entire system can be factorized, and the qubit wave function takes the form: $|\;q\left(t_{r}\right)\rangle \;=\;\alpha^{+}|\;↑\rangle \;+\;\alpha^{-}|\;↓\rangle$, where the amplitudes depend on the Rabi-frequency and pulse duration. As is well known, the complex parameters $\alpha^{+}$ and $\alpha^{-}$ determine the angles that define the orientation of the vector $s\;=\;\langle q\left(t_{r}\right)\;|\;$$\vec{\sigma }$$|q(t_{r})\rangle$ on the Bloch sphere. The probability that the z-projection of vector s has a positive value (in other words, the population of the lower level) is equal to $|\alpha^{+}{|}^{2}$, the negative projection and the population of the upper level are equal $|\alpha^{-}{|}^{2}$.

If there is a coupling between the qubit and the oscillator, λ ≠ 0, after the end of the π/2-pulse at the time $t\;=\;t_{r}$, Figure 7c, the system is in the superposition state, which already consists of the group of levels belonging to the right equilibrium point for different qubit basis states (two narrow red peaks in Figure 7c). The subsequent excitation of the measuring oscillator leads to the fact that the system at the time $t\;=\;t_{0}$ (see Figure 7d) is in the superposition of levels corresponding to the classical region on the phase space outside the separatrix with different qubit basis states σ = ±1, which corresponds to the black arrows in Figure 6a and Figure 7b–d. For the chosen typical parameters (6), the initialization time of the coupled system (qubit+sensor) is $t_{0}\;=\;50$ ns.

*Readout of qubit states by the nonlinear oscillator.* Further, we assume that the process of qubit preparation and excitation of the measuring oscillator has ended by the time $t\;=\;t_{0}$, then the general wave function is obtained as

(15) $$|\Psi (t_{0})\rangle \;=\;\sum_{j}b_{j}^{+}\;|\;\phi_{j}^{+}\rangle \;⊗\;|↑\rangle \;+\;\sum_{j}b_{j}^{-}\;|\;\phi_{j}^{-}\rangle \;⊗\;|↓\rangle .$$

Since the proposed method is based on measuring the capture probability of the oscillator by the groups of red and blue levels during process of dissipation (see Figure 4d), it is necessary to take into account the connection with the environment. We find it convenient to work in the quasi-stationary basis (10). In the case when transition rates between different levels of the system are much bigger than relaxation rates, the secular approximation is valid and we can average all rapidly oscillating terms. Note that for this approximation, off-diagonal elements do not affect the evolution of diagonal elements [45]. This makes it possible to pass to the dissipation model based on the master Equation (A10), see Appendix B. The diagonal part of the system density matrix is given by

(16) $$\rho_{d}\left(t_{0}\right)\;=\;\sum_{j}\left(P_{j}^{+}\left(t_{0}\right)|\phi_{j}^{+}\rangle \langle \phi_{j}^{+}|⊗|↑\rangle \langle ↑|\;+\;P_{j}^{-}\left(t_{0}\right)|\phi_{j}^{-}\rangle \langle \phi_{j}^{-}|⊗|↓\rangle \langle ↓|\right),$$

where $P_{j}^{+}\left(t_{0}\right)\;\equiv \;{\left|b_{j}^{+}\right|}^{2}$ and $P_{j}^{-}\left(t_{0}\right)\;\equiv \;{\left|b_{j}^{-}\right|}^{2}$. The probability of finding the qubit in the ground state is defined as $\sum_{j}P_{j}^{+}\left(t_{0}\right)\;=\;p^{+}$, where the summation occurs over all levels *j* of the oscillator, and the probability of finding the qubit in the excited state is $\sum_{j}P_{j}^{-}\left(t_{0}\right)\;=\;p^{-}$, and there is the normalization condition:

(17) $$p^{+}\;+\;p^{-}\;=\;1.$$

The capture probabilities of the measuring oscillator into dynamic equilibrium positions $P_{L}$ and $P_{R}$ correspond to the probabilities of the system being at the blue and red levels, respectively, in Figure 6a. Before reaching these groups of levels, the system from the prepared state (Figure 7d) dissipates and inevitably passes through levels lying near the separatrix energy (Figure 4d). Then, similarly to the classical case, the probability of being captured in the process of relaxation into one of the equilibrium positions does not depend on the particular prepared state [39]. It is important that the probability of leaving the level *i* per unit time $W_{i}\;\equiv \;\sum_{j\neq i}W_{ji}$ is maximum for levels outside the separatrix energy (black arrows in Figure 6b). In this case, the value of $W_{i}$ decreases as it approaches the level corresponding to the classical equilibrium point (red arrows in Figure 6b). Due to this, the high relaxation rate outside the separatrix energy levels allows us to approximately find the characteristic measurement time of the qubit state as $t_{meas}\;\approx \;1/W_{i_{s}}$, where $i_{s}$ is the number of any level near the separatrix (shown in green in Figure 6b). In the case when the qubit energy relaxation time $T_{q}$ is much longer than the measurement process time ($T_{q}\;\gg \;t_{meas}$), transitions between levels with different qubit states can be considered impossible, then the system of differential Equation (A10) splits into two independent subsystems:

(18) $$\begin{array}{c} {\dot{P}}_{i}^{+}\;=\;\sum_{j\neq i}^{+}\left(W_{ji}P_{j}^{+}\;-\;W_{ij}P_{i}^{+}\right), \end{array}$$

(19) $$\begin{array}{c} {\dot{P}}_{i}^{-}\;=\;\sum_{j\neq i}^{-}\left(W_{ji}P_{j}^{-}-W_{ij}P_{i}^{-}\right), \end{array}$$

where in Equation (18) the summation occurs only over levels with the qubit state σ = 1, and in Equation (19)–over levels with the qubit state σ = −1. Since the ratio of the capture probabilities into the left and right equilibrium points with the same qubit polarization does not depend on the values of $p^{+}$ and $p^{-}$, the capture probability of the measuring oscillator into the right equilibrium point for the prepared qubit state is

(20) $$P_{R}\;=\;p^{+}P_{R}^{+}\;+\;p^{-}P_{R}^{-},$$

where $P_{R}^{+}$ is the capture probability of the measuring oscillator into the right equilibrium point with the qubit initialized to the ground state, $P_{R}^{-}$ – to the excited state. Using Equations (17) and (20) we can get the desired probability of finding the qubit in the ground state:

(21) $$p^{+}\;=\;\frac{P_{R}\;-\;P_{R}^{-}}{P_{R}^{+}-P_{R}^{-}}.$$

Thus, by measuring the $P_{R}$ value of the Josephson oscillator, it becomes possible to measure the qubit populations in the prepared state. In this case, the values $P_{R}^{+}$ and $P_{R}^{-}$ can be determined during the calibration of the measuring oscillator for various states of the qubit with the quantum number σ = ±1. To determine the capture probability of the oscillator into the right equilibrium point $P_{R}$, it is necessary, as in the experiments [14,16,17,23], to measure the phase difference between the reflected and the applied signal $I_{ex}\left(t\right)$. The frequentist probability of this phase difference corresponding to *R* branches in Figure 2d will determine the desired probability $P_{R}$. Note that the proposed method does not require changes in the experimental technique.

**Figure 8:**
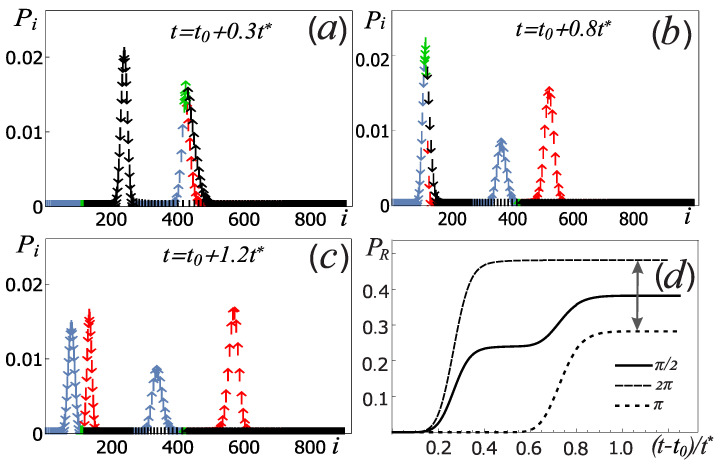
(**a**–**c**) Time evolution of the diagonal elements of the system density matrix $P_{i}$ in the process of the qubit measurement at different times. (**d**) The dependence of the capture probability into the right equilibrium point $P_{R}$ as a function of time for different Rabi pulses. At the initial moment of time, the qubit was initialized to the ground state. The use of colored arrows is the same as in Figure 6a,b. The system parameters used are determined by the ratios (6).

Figure 8 shows the simulation of the measurement process at $t\;>\;t_{0}$, as a result of which the system (Figure 7d) dissipates due to the connection with the bosonic thermostat, passing into a mixed state consisting of groups of levels belonging to different levels of the measuring oscillator and with different qubit states. Figure 8a–c shows the population probabilities of the system levels $P_{i}$ at different times, which were obtained by numerically solving (A10). In numerical calculations, $t^{*}\;=\;\frac{\hbar^{2}}{2\pi g\kappa^{2}}$ is used as the unit of time. As can be seen from Figure 8a–c, in the process of evolution, a redistribution takes place among the levels in the system. After passing through the group of levels corresponding to the separatrix energy (marked in green), the qubit states are separated (in the time $t_{meas}$∼$1.2t^{*}$). Since the probabilities $P_{R}$ and $P_{L}$ will not change during the further evolution of the system even if the coherence of the qubit states is broken, the measuring oscillator can function as a memory device. Note that the different positions of the Lorentzian peaks in Figure 8c are related to the level numbering; in the coordinate representation, the centers of these peaks will be close to each other due to the small coupling coefficient of qubit and oscillator [33]. Using the value of the quality factor of the measuring oscillator Q, one can obtain an estimate of the measurement time $t_{meas}$∼$\frac{Q}{\omega_{0}}$, which gives $t_{meas}$∼100 ns for Q∼100, according to [46]. Therefore, we get that the system initialization process (qubit and measuring oscillator) and the measurement process is $t_{0}\;+\;t_{meas}$∼150 ns, which according to [2] is much less than typical relaxation and dephasing times in the systems we are studying.

To determine the probability of the $P_{R}$ capturing, it is necessary to sum the probabilities $P_{i}$ of the entire group of levels (red arrows in Figure 8a–c) at time $t_{meas}$ related to the right equilibrium point for different qubit states. Figure 8d shows the dependence of $P_{R}$ on time for various pulses. It can be seen that this capture probability reaches a constant value after the system passes through a group of levels lying near the separatrix energy in the process of relaxation. It follows from the analysis that using the proposed scheme of the initialization (Figure 7) and the measurement (Figure 8a–c), according to (21) it is possible to determine the probabilities of finding the qubit in the basic states. Neglecting the back action, the Δ value, in substance, determines the distance indicated by the gray arrow in Figure 8d. So, for example, for the π/2-pulse with the chosen parameters of the systems, we found that the probability of finding the qubit in the ground state is $p^{+}\;=\;0.5003$. The difference of this probability from 0.5 is due to the influence of the measuring oscillator on the qubit during initialization (back action effect), when the states of the subsystems were being entangled.

## 5. Conclusions

The new method for measuring the qubit states by the nonlinear Josephson oscillator is proposed. It is based on the sensitivity of the oscillator capture probabilities into dynamic equilibrium depending on the states of the qubit. This is due to the sensitivity of the transition rates of levels near the separatrix energy in the quantum regime to the qubit state, which in the classical regime is similar to the sensitivity of different separatrix lobes areas. The measurement is nondemolition because after the qubit initialization (ϵ(t) = 0) the interaction operator commutes with the system Hamiltonian: $[H_{int},\;H]\;=\;0$. We have found the drive current parameters for which the separation efficiency for different qubit states is the maximum. The back action of the measuring oscillator on the qubit was analyzed within the framework of the microwave approach. In order to control a qubit with the required fidelity, the measuring oscillator should be prepared in a superposition of states with small average occupation numbers. The optimal amplitude of control pulses to achieve the best fidelity: the Rabi frequency is equal to the natural frequency of the measuring oscillator.

The measurement protocol using the high-Q bifurcation oscillator for state populations of the superconducting qubit has been developed. Note that in this approach the considered sensor can be used as a memory device. This is possible because, after the measurement, the oscillator is in a superposition of states with energies far from the energy of the classical separatrix. At the same time, the transition probabilities between groups of levels related to different equilibrium points due to external influences are negligible.

We have obtained an estimate of the time of initialization and measurement of states for typical parameters [16,24] for “qubit+oscillator” system: ∼150 ns. Since the proposed method can be used in the parameter range where bifurcation behavior exists for both qubit basis states, an additional so-called “latched section” [16] in the readout current pulse is not required. We hope that this feature will simplify the process and reduce the measurement time. Thus, we have demonstrated that the high-Q measuring oscillator can play a role of the sensor in the parameter range where bifurcation behavior exists for both qubit basis states. We expect this method to be applicable to other types of qubits such as transmons [3].

## Data Availability

Data are contained within the article.

## Appendix A

To obtain (5), we use the generating function

(A1) $$F\left(\varphi ,\;q,\;t\right)\;=\;\left(\varphi^{2}\frac{\omega_{0}C\Phi_{0}^{2}}{2\hbar }\;+\;\frac{q^{2}}{2}\right)\mathrm{ctg}\left(\omega t\right)\;-\;\varphi q\sqrt{\frac{\omega_{0}C\Phi_{0}^{2}}{\hbar }}\frac{1}{\sin(\omega t)}$$

with the valence c = 1/ℏ to perform the canonical transformation $\left(\varphi ,\;p_{\varphi }\right)\;\to \;\left(q,\;p\right)$, where the old momentum $p_{\varphi }\;=\;C\Phi_{0}^{2}\dot{\varphi }$. The equations defining the transformations are written as

(A2) $$\begin{array}{ccc} p_{\varphi } & = & \frac{1}{c}\frac{\partial F}{\partial \varphi }, \end{array}$$

(A3) $$\begin{array}{ccc} p & = & -\frac{\partial F}{\partial q}, \end{array}$$

(A4) $$\begin{array}{ccc} H_{osc}\left(q,p\right) & = & cH_{osc}\left(\varphi ,p_{\varphi }\right)\;+\;\frac{\partial F}{\partial t}. \end{array}$$

It’s easy to obtain old canonical coordinates as functions of the new ones as

(A5) $$\begin{array}{ccc} \varphi & = & \sqrt{\frac{\hbar }{\omega_{0}C\Phi_{0}^{2}}}\left(p\sin\omega t\;+\;q\cos\omega t\right), \end{array}$$

(A6) $$\begin{array}{ccc} p_{\varphi } & = & \sqrt{\hbar \omega_{0}C\Phi_{0}^{2}}\left(p\cos\omega t\;-\;q\sin\omega t\right). \end{array}$$

Consequently, we obtain the Hamiltonian for new canonical coordinates as

(A7) $$\begin{array}{c} H_{osc}\left(q,\;p\right)\;=\;\left(\omega_{0}\;-\;\omega \right)\left(\frac{q^{2}}{2}\;+\;\frac{p^{2}}{2}\right)\;-\;\frac{\hbar }{16C\Phi_{0}^{2}}{\left(p\sin\omega t\;+\;q\cos\omega t\right)}^{4}\;-\;\frac{I_{0}}{\sqrt{\hbar \omega_{0}C}}\cos\omega t\left(p\sin\omega t\;+\;q\cos\omega t\right). \end{array}$$

In accordance with the slowly varying amplitude approximation, we can average the resulting Hamiltonian over the period 2π/ω. Introducing new time τ = ℏt, we finally obtain (5).

In the quantum regime, we use the unitary transformation $U\;=\;e^{i\omega \hat{a^{†}}\hat{a}t}$ to the Hamiltonian (8)

(A8) $$\begin{array}{c} H_{RWA}\;=\;UHU^{†}\;-\;\hbar \omega \hat{a^{†}}\hat{a}. \end{array}$$

Similarly, averaging this Hamiltonian over the period, we obtain (9) in the rotating wave frame.

## Appendix B

Due to the fact that the nonlinear oscillator measuring qubit states interacts with the environment, an important issue is to take into account the dissipation on the measurement process. We consider a model of bosonic bath linearly connected to subsystems:

(A9) $$V\;=\;\kappa \left(a^{†}\;+\;a\right)\sum_{k}\left(B_{k}^{†}\;+\;B_{k}\right)\;+\;\kappa_{q}r\sum_{k}\left(B_{k}^{†}\;+\;B_{k}\right),$$

where the operator $r\;=\;\frac{1}{2}\left(1\;-\;\sigma_{x}\right)$ is responsible for the energy relaxation of the qubit [47], ${B_{k}}^{†}$ and $B_{k}$ are the operators of the creation and annihilation of bosonic oscillators, κ and $\kappa_{q}$ are the coupling coefficients of bosonic modes with the measuring oscillator and the qubit, respectively. We use dissipation model in which the density of bosonic modes *g* and coupling coefficients do not depend on the mode number. The equation for the subsystem density matrix in the Born-Markov and secular approximations, after averaging over the states of the bosonic bath, it is possible to reduce to the master equation for diagonal elements $P_{i}\left(t\right)\;=\;\rho_{ii}\left(t\right)$ [39]. The equation in the eigenbasis of the Hamiltonian (9) may be written as

(A10) $${\dot{P}}_{i}\;=\;\sum_{j\neq i}\left(W_{ji}P_{j}\;-\;W_{ij}P_{i}\right),$$

where are the designations introduced:

$$\begin{array}{cc} & W_{ji}\;\equiv \;\frac{2\pi }{\hbar^{2}}(\kappa^{2}\left(\right|a_{ji}{|}^{2}{\bar{W}}_{ji}\;+\;|a_{ij}{|}^{2}{\bar{W}}_{ij})\;+\;\kappa_{q}^{2}|r_{ji}{|}^{2}\left(g\left(\omega_{ji}\right)\left(\bar{n}\left(\omega_{ji}\right)\;+\;1\right)\;+\;g\left(\omega_{ij}\right)\bar{n}\left(\omega_{ij}\right)\right)),\\ & {\bar{W}}_{ji}\;\equiv \;g\left(\omega \;-\;\omega_{ji}\right)\left(\bar{n}\left(\omega \;-\;\omega_{ji}\right)\;+\;1\right)\;+\;g\left(\omega_{ji}\;-\;\omega \right)\bar{n}\left(\omega_{ji}\;-\;\omega \right),\\ & {\bar{W}}_{ij}\;\equiv \;g\left(\omega_{ij}\;-\;\omega \right)\left(\bar{n}\left(\omega_{ij}\;-\;\omega \right)\;+\;1\right)\;+\;g\left(\omega \;-\;\omega_{ij}\right)\bar{n}\left(\omega \;-\;\omega_{ij}\right),\\ & a_{ji}\;\equiv \;\langle \psi_{j}|a\;|\;\psi_{i}\rangle ,\;r_{ji}\;\equiv \;\langle \psi_{j}|r\;|\;\psi_{i}\rangle ,\;\omega_{ji}\;\equiv \;\frac{E_{j}-E_{i}}{\hbar }. \end{array}$$

It is assumed here that the arguments for the density of bosonic modes *g* and for Planck distribution $\bar{n}$ must be positive. $W_{ji}$ is the transition rate between different system states (10): from $|\;\psi_{i}\rangle$ to $|\;\psi_{j}\rangle$.

## References

[1] Blais A., Huang R.-S., Wallraff A., Girvin S.M., Schoelkopf R.J. (2004). Cavity quantum electrodynamics for superconducting electrical circuits: An architecture for quantum computation. Phys. Rev. A.

[2] Kjaergaard M., Schwartz M.E., Braumüller J., Krantz P., Wang J., Gustavsson S., Oliver W.D. (2020). Superconducting qubits: Current state of play. Annu. Rev. Condens. Matter Phys..

[3] Krantz P., Kjaergaard M., Yan F., Orlando T.P., Gustavsson S., Oliver W.D. (2019). A Quantum Engineer’s Guide to Superconducting Qubits. Appl. Phys. Rev..

[4] Gu X., Kockum A.F., Miranowicz A., Liu Y., Nori F. (2017). Microwave photonics with superconducting quantum circuits. Phys. Rep..

[5] Vozhakov V.A., Bastrakova M.V., Klenov N.V., Soloviev I.I., Pogosov W.V., Babukhin D.V., Zhukov A.A., Satanin A.M. (2022). State control in superconducting quantum processors. Phys.-Uspekhi.

[6] Walter T., Kurpiers P., Gasparinetti S., Magnard P., Potočnik A., Salathé Y., Pechal M., Mondal M., Oppliger M., Eichler C. (2017). Rapid High-Fidelity Single-Shot Dispersive Readout of Superconducting Qubits. Phys. Rev. Appl..

[7] Chen L., Li H.-X., Lu Y., Warren C.W., Križan C.J., Kosen S., Rommel M., Ahmed S., Osman A., Biznárová J. (2022). Transmon qubit readout fidelity at the threshold for quantum error correction without a quantum-limited amplifier. arXiv.

[8] Simoen M.C., Chang W.S., Krantz P., Bylander J., Wustmann W., Shumeiko V., Delsing P., Wilson C.M. (2015). Characterization of a multimode coplanar waveguide parametric amplifier. J. Appl. Phys..

[9] Sivak V.V., Shankar S., Liu G., Aumentado J., Devoret M.H. (2020). Josephson array-mode parametric amplifier. Phys. Rev. Appl..

[10] Roch N., Schwartz M.E., Motzoi F., Macklin C., Vijay R., Eddins A.W., Korotkov A.N., Whaley K.B., Sarovar M., Siddiqi I. (2014). Observation of measurement-induced entanglement and quantum trajectories of remote superconducting qubits. Phys. Rev. Lett..

[11] Sun L., Petrenko A., Leghtas Z., Vlastakis B., Kirchmair G., Sliwa K.M., Narla A., Hatridge M., Shankar S., Blumoff J. (2014). Tracking photon jumps with repeated quantum non-demolition parity measurements. Nature.

[12] Eddins A., Kreikebaum J.M., Toyli D.M., Levenson-Falk E.M., Dove A., Livingston W.P., Levitan B.A., Govia L.C.G., Clerk A.A., Siddiqi I. (2019). High-Efficiency Measurement of an Artificial Atom Embedded in a Parametric Amplifier. Phys. Rev. X.

[13] Mallet F., Ong F.R., Palacios-Laloy A., Nguyen F., Bertet P., Vion D., Esteve D. (2009). Single-shot qubit readout in circuit quantum electrodynamics. Nat. Phys..

[14] Vijay R., Slichter D.H., Siddiqi I. (2011). Observation of Quantum Jumps in a Superconducting Artificial Atom. Phys. Rev. Lett..

[15] Siddiqi I., Vijay R., Pierre F., Wilson C.M., Metcalfe M., Rigetti C., Frunzio L., Devoret M.H. (2004). RF-Driven Josephson Bifurcation Amplifier for Quantum Measurement. Phys. Rev. Lett..

[16] Siddiqi I., Vijay R., Metcalfe M., Boaknin E., Frunzio L., Schoelkopf R.J., Devoret M.H. (2006). Dispersive measurements of superconducting qubit coherence with a fast latching readout. Phys. Rev. B.

[17] Lupascu A., Saito S., Picot T., de Groot P.C., Harmans C.J.P.M., Mooij J.E. (2007). Quantum non-demolition measurement of a superconducting two-level system. Nat. Phys..

[18] Vijay R., Devoret M.H., Siddiqi I. (2009). Invited review article: The Josephson bifurcation amplifier. Rev. Sci. Instrum..

[19] Schmitt V., Zhou X., Juliusson K., Royer B., Blais A., Bertet P., Vion D., Esteve D. (2014). Multiplexed readout of transmon qubits with Josephson bifurcation amplifiers. Phys. Rev. A.

[20] Zorin A.B., Makhlin Y. (2011). Period-doubling bifurcation readout for a Josephson qubit. Phys. Rev. B.

[21] Mahashabde S., Otto E., Montemurro D., de Graaf S., Kubatkin S., Danilov A. (2020). Fast Tunable High-Q-Factor Superconducting Microwave Resonators. Phys. Rev. Appl..

[22] Budoyo R.P., Kakuyanagi K., Toida H., Matsuzaki Y., Munro W.J., Yamaguchi H., Saito S. (2018). Electron paramagnetic resonance spectroscopy of Er3+:Y2SiO using a Josephson bifurcation amplifier: Observation of hyperfine and quadrupole structures. Phys. Rev. Mater..

[23] Budoyo R.P., Kakuyanagi K., Toida H., Matsuzaki Y., Munro W.J., Saito S. (2020). Electron spin resonance with up to 20 spin sensitivity measured using a superconducting flux qubit. Appl. Phys. Lett..

[24] Schaal S., Ahmed I., Haigh J.A., Hutin L., Bertrand B., Barraud S., Vinet M., Lee C.-M., Stelmashenko N., Robinson J.W.A. (2020). Fast Gate-Based Readout of Silicon Quantum Dots Using Josephson Parametric Amplification. Phys. Rev. Lett..

[25] Shelly C.D., Checkley C., Petrashov V.T. (2021). Hybrid Quantum Interferometer in Bifurcation Mode as a Latching Quantum Readout. Phys. Rev. App..

[26] Dykman M. (2007). Critical exponents in metastable decay via quantum activation. Phys. Rev. E.

[27] Nakano H., Saito S., Semba K., Takayanagi H. (2009). Quantum Time Evolution in a Qubit Readout Process with a Josephson Bifurcation Amplifier. Phys. Rev. Lett..

[28] Denisenko M.V., Munyayev V.O., Satanin A.M. (2016). Quantum fractional resonances in superconducting circuits with an embedded Josephson junction. J. Phys. Conf. Ser..

[29] Denisenko M.V., Munyaev V.O., Satanin A.M. (2016). Parametric frequency transformation in a superconducting waveguide line with an integrated Josephson oscillator. Phys. Solid State.

[30] Gergel’ V.P., Denisenko M.V., Linev A.V., Satanin A.M. (2016). Selective measurements of superconducting qubit states by a nonlinear Josephson oscillator. Phys. Solid State.

[31] Gosner J., Kubala B., Ankerhold J. (2019). Quantum properties of a strongly driven Josephson junction. Phys. Rev. B.

[32] Chang C.W.S., Sabín C., Forn-Díaz P., Quijandría F., Vadiraj A.M., Nsanzineza I., Johansson G., Wilson C.M. (2020). Observation of three-photon spontaneous para-metric down-conversion in a superconducting parametric cavity. Phys. Rev. X.

[33] Pashin D.S., Bastrakova M.V. (2020). Bistable Josephson cell as a single microwave photon sensor. Int. J. Quantum Inf..

[34] Di Candia R., Minganti F., Petrovnin G.S., Paraoanu G.S., Felicetti S. (2021). Critical parametric quantum sensing. arXiv.

[35] Arndt L., Hassler F. (2022). Period Tripling due to Parametric Down-Conversion in Circuit QED. Phys. Rev. Lett..

[36] Dykman M. (2012). Fluctuating Nonlinear Oscillators: From Nanomechanics to Quantum Superconducting Circuits.

[37] Vion D., Aassime A., Cottet A., Joyez P., Pothier H., Urbina C., Esteve D., Devoret M.H. (2002). Manipulating the Quantum State of an Electrical Circuit. Science.

[38] Bogoliubov N.N., Mitropolsky Y.A. (1961). Asymptotic Methods in the Theory of Non-Linear Oscillations.

[39] Pashin D., Satanin A.M., Kim C.S. (2019). Classical and quantum dissipative dynamics in Josephson junctions: An Arnold problem, bifurcation, and capture into resonance. Phys. Rev. E.

[40] Arnold V.I. (1963). Small denominators and problems of stability of motion in classical and celestial mechanics. Usp. Mat. Nauk..

[41] Kim C.S., Pashin D.S., Satanin A.M. (2017). Quantum dissipative dynamics a particle in a double-well potential. Lobachevskii J. Math..

[42] Bowdrey M.D., Oi D.K.L., Short A.J., Banaszek K., Jones J.A. (2002). Fidelity of single qubit maps. Phys. Lett. A.

[43] Ekert A., Knight P.L. (1995). Entangled Quantum-Systems and the Schmidt Decomposition. Am. J. Phys..

[44] Pashin D., Bastrakova M.V., Satanin A.M., Kim C.S. (2020). Quantum analog of bifurcation and switching effects in a nonlinear Josephson oscillator. AIP Conf. Proc..

[45] Blum K. (1981). Density Matrix Theory and Applications.

[46] Manucharyan V.E., Boaknin E., Metcalfe M., Vijay R., Siddiqi I., Devoret M. (2007). Microwave bifurcation of a Josephson junction: Embedding-circuit requirements. Phys. Rev. B.

[47] Makhlin Y., Schön G., Shnirman A., Fazio R., Gantmakher V.F., Imry Y. (2003). Dissipation in Josephson qubits. New Directions in Mesoscopic Physics (Towards Nanoscience).

